# Supplementary material for: Structure of an RNA polymerase ribozyme replication complex
Source: bioRxiv. 2026 Aug 31:2026.08.30.748161. Preprint. [Version 1] doi: 10.64898/2026.08.30.748161 (PMC13684603; doi:10.64898/2026.08.30.748161)
Supplement: Supplementary file 1 [file NIHPP2026.08.30.748161v1-supplement-1.pdf]

bioRxiv preprint doi: https://doi.org/10.64898/2026.08.30.748161; this version posted August 31, 2026. The copyright holder for this preprint (which was not certified by peer review) is the author/funder, who has granted bioRxiv a license to display the preprint in perpetuity. It is made available under a CC-BY 4.0 International license.

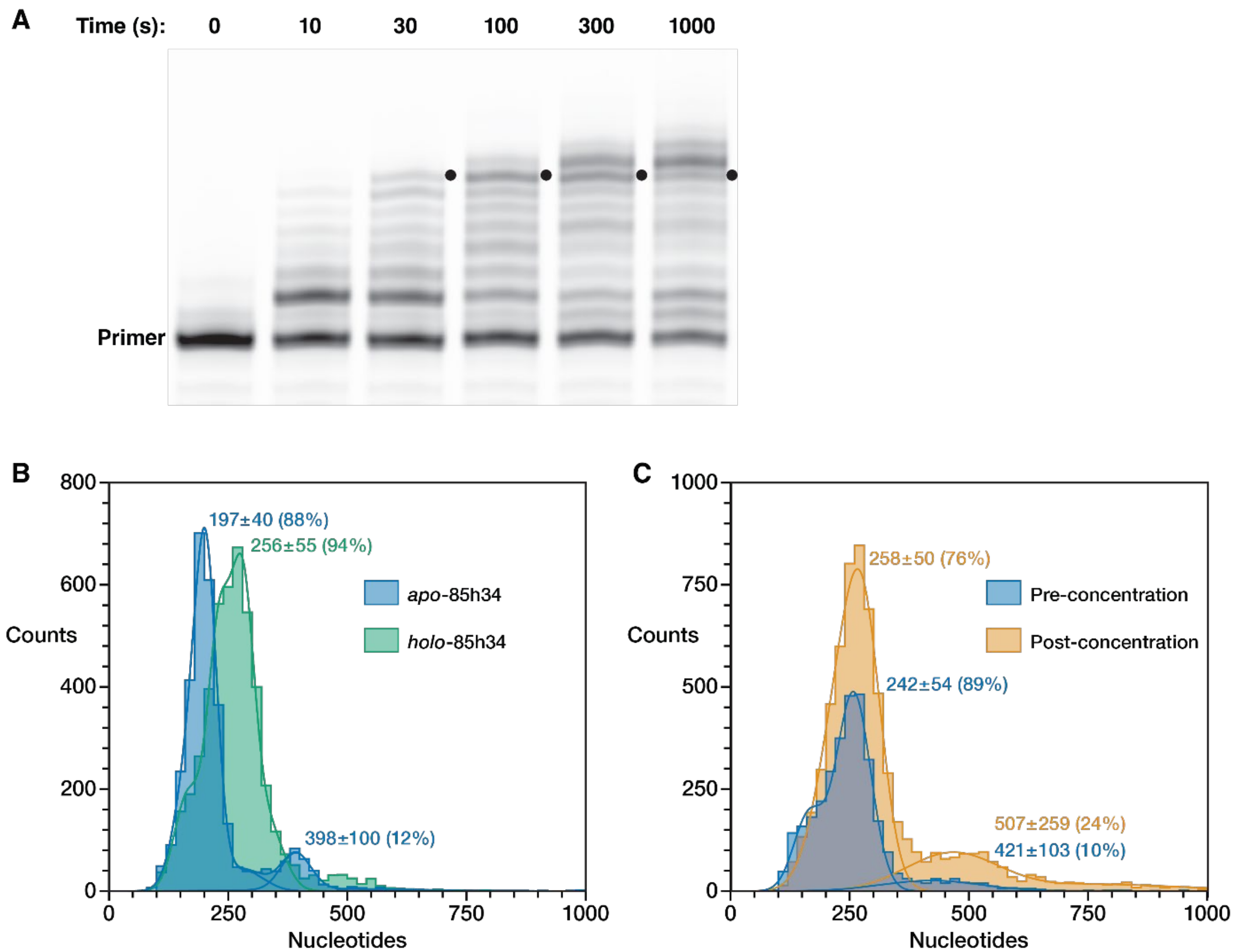

Fig. S1.

Biochemical characterization of 85h34. (**A**) Activity of the 85h34 polymerase, extending 5´-fluorescein-labeled primer P1 on template T1 in the presence of 2 mM each NTP and 50 mM $MgCl_2$ at pH 8.3 and 25 °C. The extension products were separated by PAGE. Black dots indicate full-length products. (**B**) Replication complexes were assembled using 85h34, P1, T1 and AMPcPP and analyzed by mass photometry. The complex peak is compared to 85h34 alone. (**C**) The assembled complexes were concentrated 10-fold using a spin-concentration device, then re-analyzed by mass photometry prior to vitrification for cryo-EM analysis. The expected sizes of monomeric *apo*- and *holo*-85h34 are 194 and 257 nucleotides, respectively. Individual peaks are labeled with the calculated mean and standard deviation of the nucleotide content based on a Gaussian fit of the data, together with the fraction of the population in that peak.

bioRxiv preprint doi: https://doi.org/10.64898/2026.08.30.748161; this version posted August 31, 2026. The copyright holder for this preprint (which was not certified by peer review) is the author/funder, who has granted bioRxiv a license to display the preprint in perpetuity. It is made available under a CC-BY 4.0 International license.

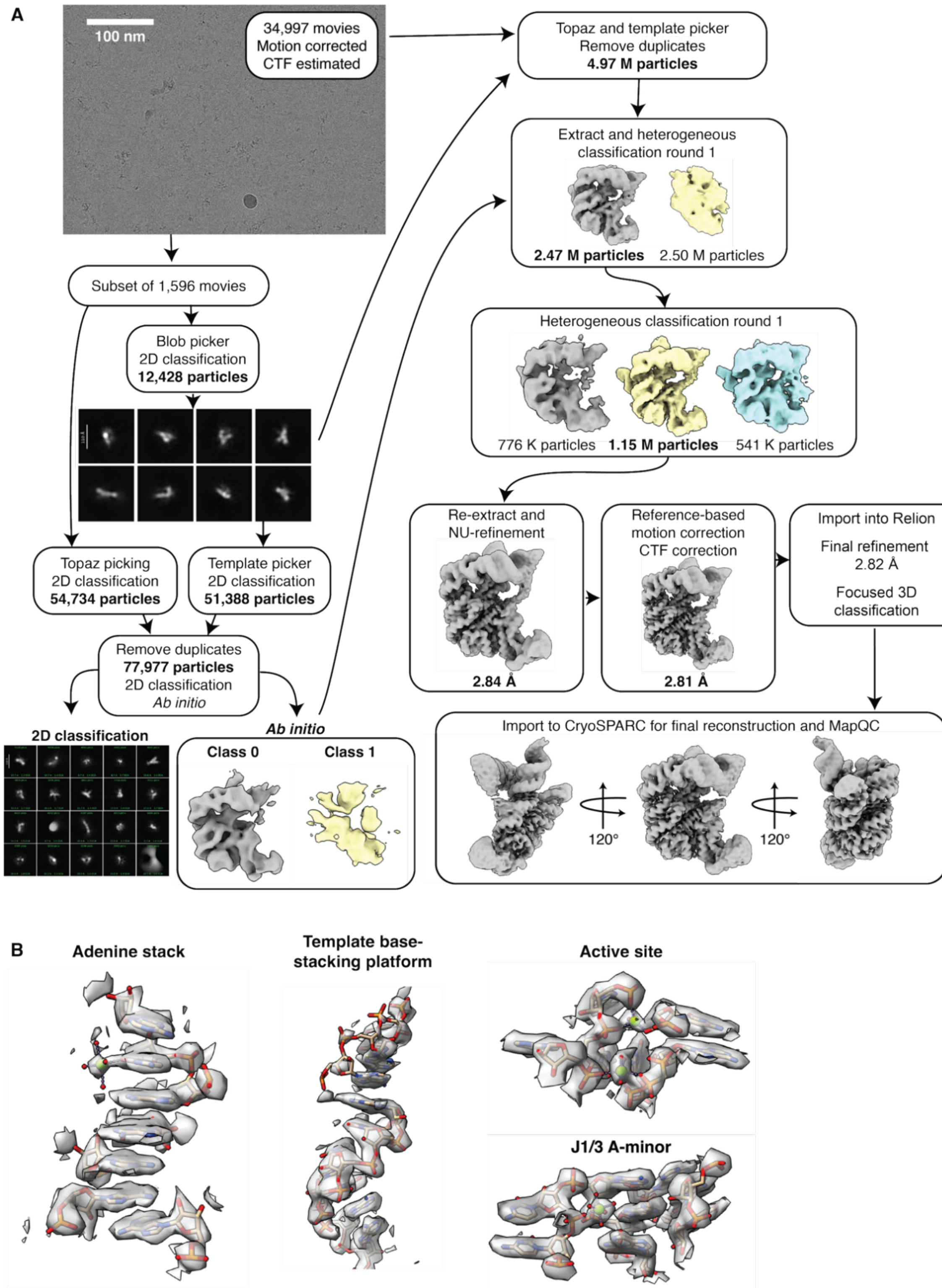

Fig. S2.

Cryo-EM data processing scheme. (**A**) Representative micrographs, 2D classification results, and representative coulombic potential maps. (**B**) Snapshots of models fitted within the sharpened map.

bioRxiv preprint doi: https://doi.org/10.64898/2026.08.30.748161; this version posted August 31, 2026. The copyright holder for this preprint (which was not certified by peer review) is the author/funder, who has granted bioRxiv a license to display the preprint in perpetuity. It is made available under a CC-BY 4.0 International license.

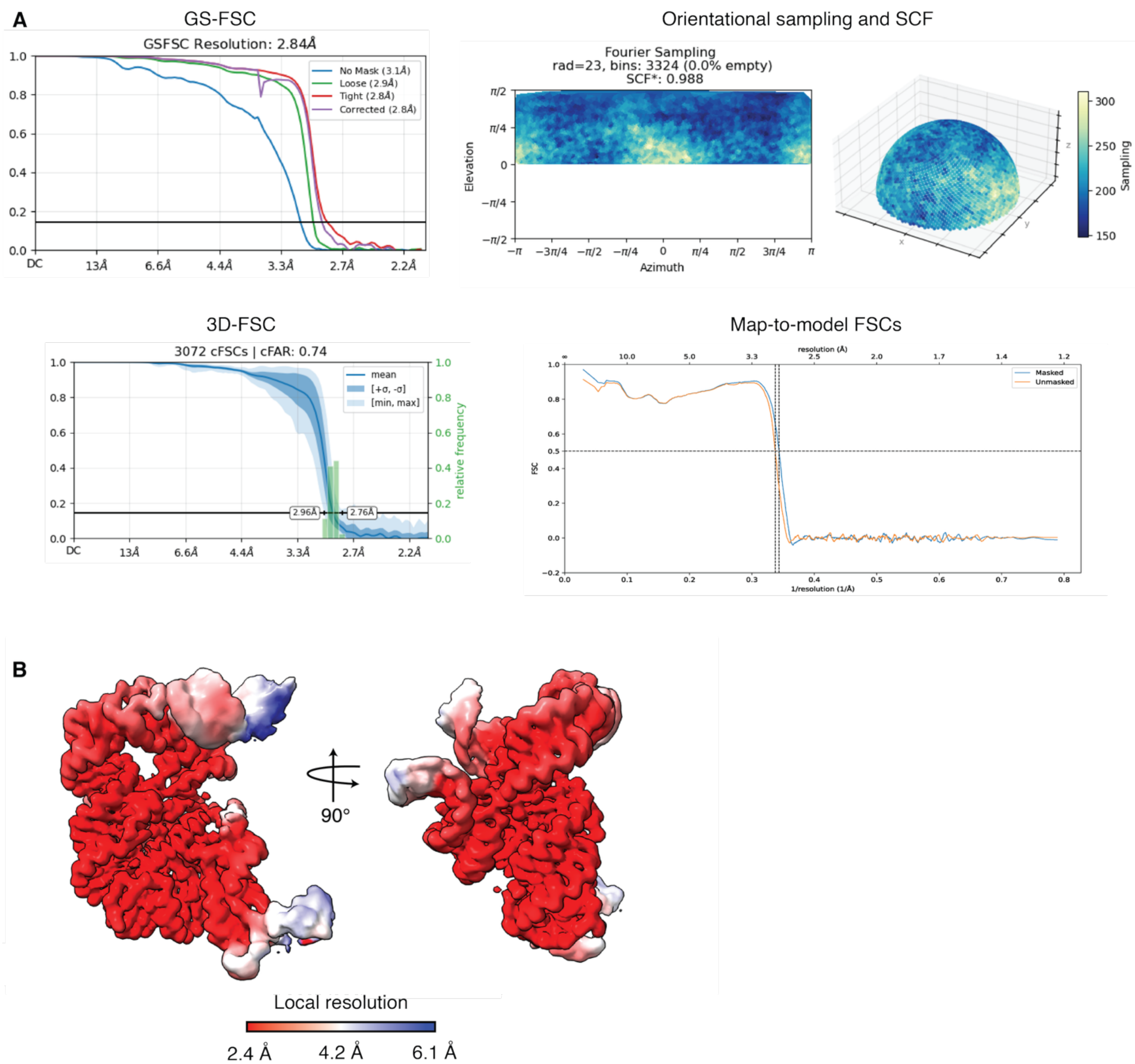

Fig. S3.

Cryo-EM map quality control measurements. (**A**) Grid showing gold standard Fourier shell correlations (GS-FSC), 3D conical FSCs (3D-FSC), orientational sampling as depicted as an Euler plot and on a half globe, sampling compensation factor and empty Fourier bins calculations, and map-to-model FSCs. (**B**) Local resolution was painted onto the coulombic potential map according to the color key.

bioRxiv preprint doi: https://doi.org/10.64898/2026.08.30.748161; this version posted August 31, 2026. The copyright holder for this preprint (which was not certified by peer review) is the author/funder, who has granted bioRxiv a license to display the preprint in perpetuity. It is made available under a CC-BY 4.0 International license.

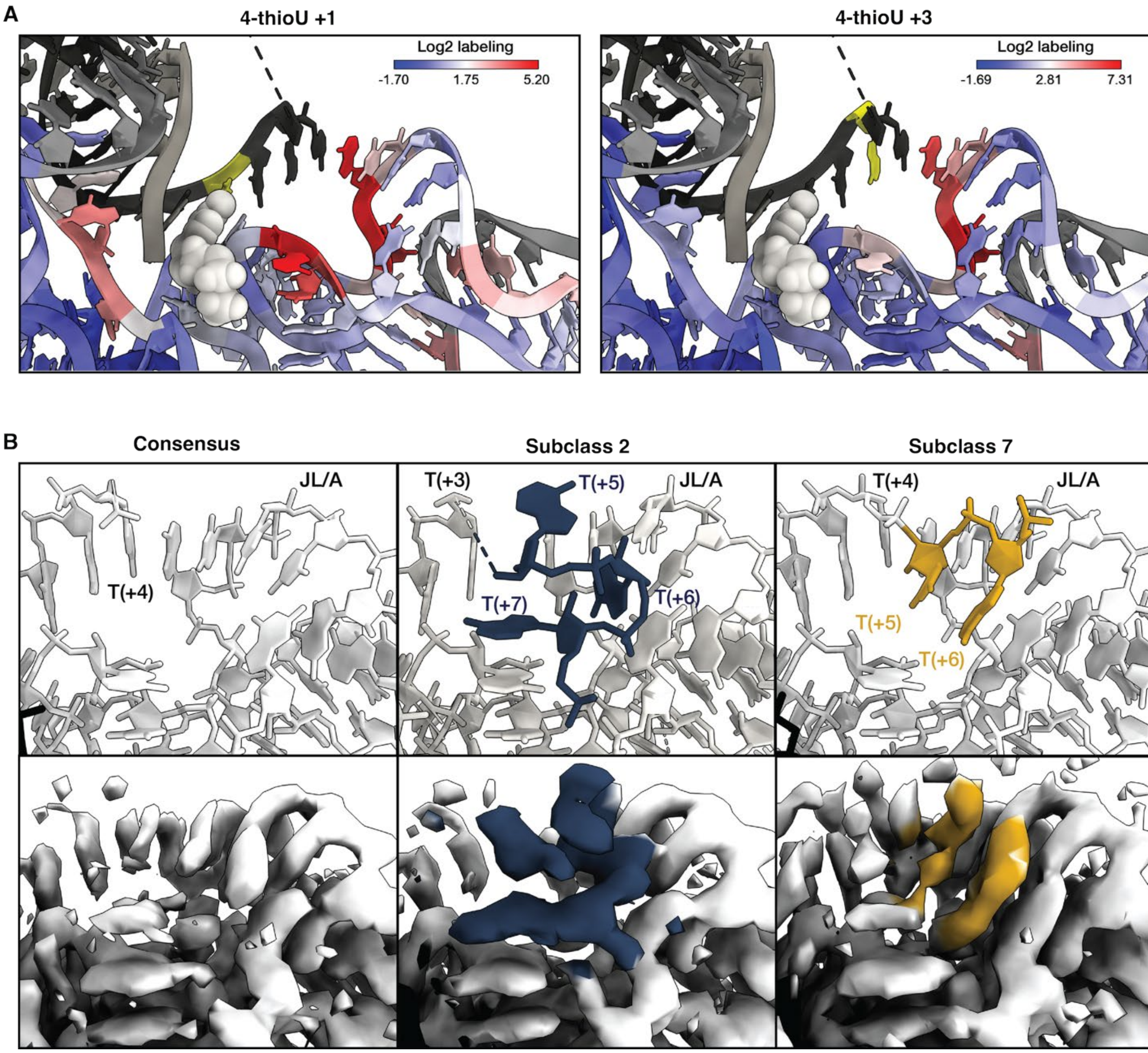

Fig. S4.

Tertiary contacts between the template and accessory domain. (**A**) Labeling of cross-linked positions in the ribozyme based on a previous study in which 4-thioU was installed at either the +1 or +3 position of the template and UV-induced cross-links were identified by stalling of reverse transcriptase (*15*). Cross-linking efficiency at various nucleotide positions is shown on a $\log_2$ scale. Positions without values are shown in gray. The position where 4-thioU was installed is colored yellow. AMPcPP is shown as space filling atoms, colored white. (**B**) Structural detail at the site of cross-linking, comparing the consensus map and two other subclasses that were identified by focused 3D classification (fig. S5). Significant heterogeneity is seen in the downstream portion of the template (positions +4 to +7) and the base-stacking platform within the JL/A loop.

bioRxiv preprint doi: https://doi.org/10.64898/2026.08.30.748161; this version posted August 31, 2026. The copyright holder for this preprint (which was not certified by peer review) is the author/funder, who has granted bioRxiv a license to display the preprint in perpetuity. It is made available under a CC-BY 4.0 International license.

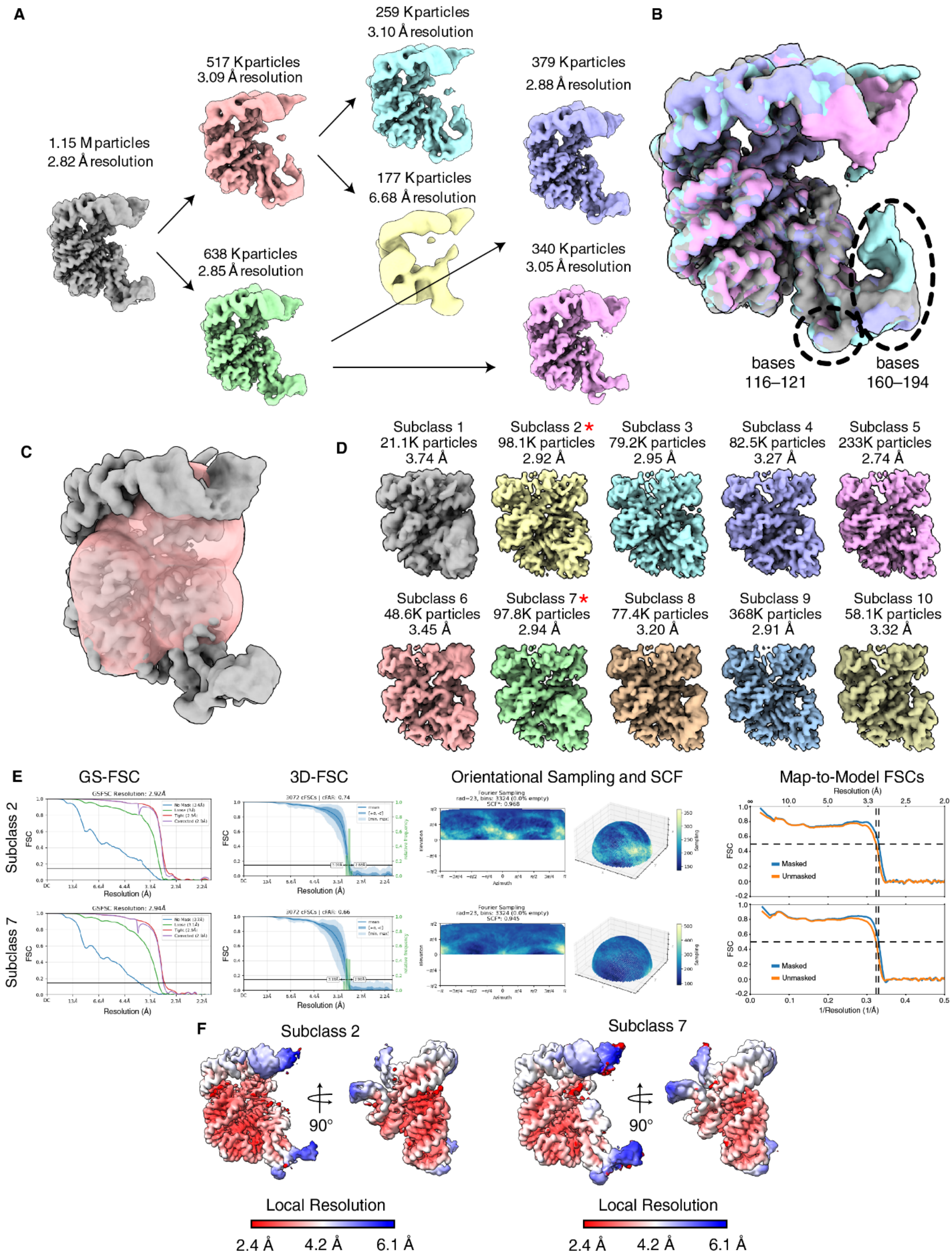

bioRxiv preprint doi: https://doi.org/10.64898/2026.08.30.748161; this version posted August 31, 2026. The copyright holder for this preprint (which was not certified by peer review) is the author/funder, who has granted bioRxiv a license to display the preprint in perpetuity. It is made available under a CC-BY 4.0 International license.

Fig. S5.

Heterogeneity analysis of consensus particle stack. (**A**) Global heterogeneity was assessed using iterative 3D classification with alignment into two classes. (**B**) Resulting maps are overlayed with the consensus map. (**C**) Masking strategy focusing on the active site for focused 3D classification without alignment. (**D**) 3D classification results for 10 classes are shown. The resulting maps did not reveal any notable differences. However, subclasses 2 and 7 (red star) contain additional density corresponding to downstream portions of the template strand, which could reliably be interpreted with an atomic model and is supported by previous cross-linking studies (fig. S4) (*15*). Although subclass 2 was nominally resolved to 2.74 Å, which is higher than the consensus map, it did not reveal any additional features within the active site. Because the consensus map showed more evenly distributed resolution, it was used for all analyses in this study. (**E**) Grid showing gold standard Fourier shell correlations (GS-FSC), 3D conical FSCs (3D-FSC), orientational sampling as depicted as an Euler plot and on a half globe, sampling compensation factor and empty Fourier bins calculations, and map-to-model FSCs. (**F**) Local resolution was painted onto the coulombic potential map according to the color key.

bioRxiv preprint doi: https://doi.org/10.64898/2026.08.30.748161; this version posted August 31, 2026. The copyright holder for this preprint (which was not certified by peer review) is the author/funder, who has granted bioRxiv a license to display the preprint in perpetuity. It is made available under a CC-BY 4.0 International license.

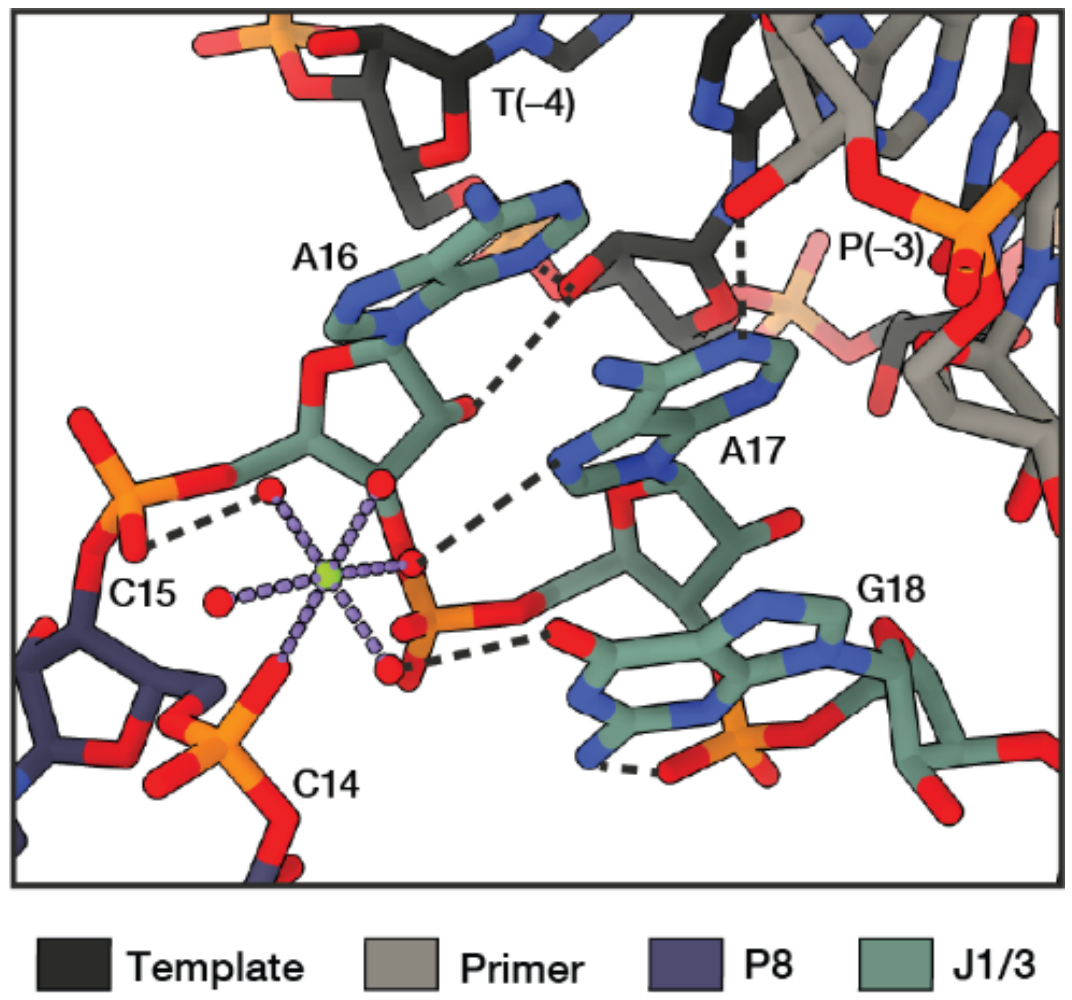

Fig. S6.

J1/3 A-minor tertiary contacts with the primer-template. Atomic model of J1/3 A-minor interactions, with hydrogen bonds and metal coordination shown as dashed black and purple lines, respectively.

bioRxiv preprint doi: https://doi.org/10.64898/2026.08.30.748161; this version posted August 31, 2026. The copyright holder for this preprint (which was not certified by peer review) is the author/funder, who has granted bioRxiv a license to display the preprint in perpetuity. It is made available under a CC-BY 4.0 International license.

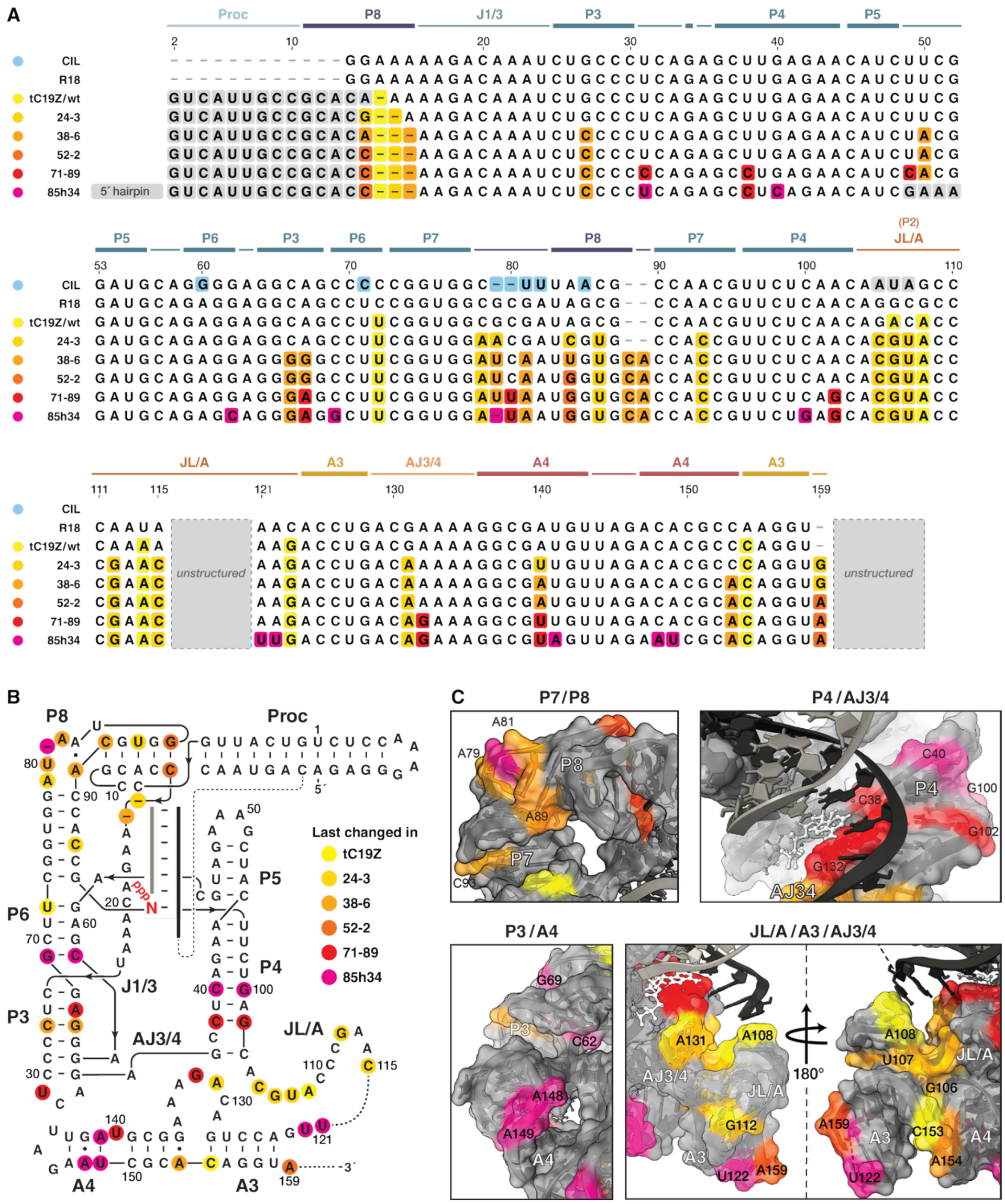

Fig. S7.

Structural analysis of the mutational landscape. (**A**) Sequence alignment of 85h34 and ancestral polymerase ribozymes: the class I ligase (CIL) (*5*, *6*); the first polymerase ribozyme, R18 (*6*); the first polymerase to contain the processivity tag, tC19Z (*8*); and polymerases from rounds 24, 38, 52, 71 in the evolutionary lineage from tC19Z to 85h34 (*9*–*12*), The tC19Z sequence includes mutations introduced at the outset of the 85h34 lineage (*9*). Mutations are color coded based on the generation of evolution in which they first appeared. (**B**) Mutations are highlighted in the secondary structure diagram and (**C**) painted onto snapshots of the structure of the 85h34 replication complex. Where multiple mutations occurred at the same site, mutations are colored by the last change that occurred at the position.

bioRxiv preprint doi: https://doi.org/10.64898/2026.08.30.748161; this version posted August 31, 2026. The copyright holder for this preprint (which was not certified by peer review) is the author/funder, who has granted bioRxiv a license to display the preprint in perpetuity. It is made available under a CC-BY 4.0 International license.

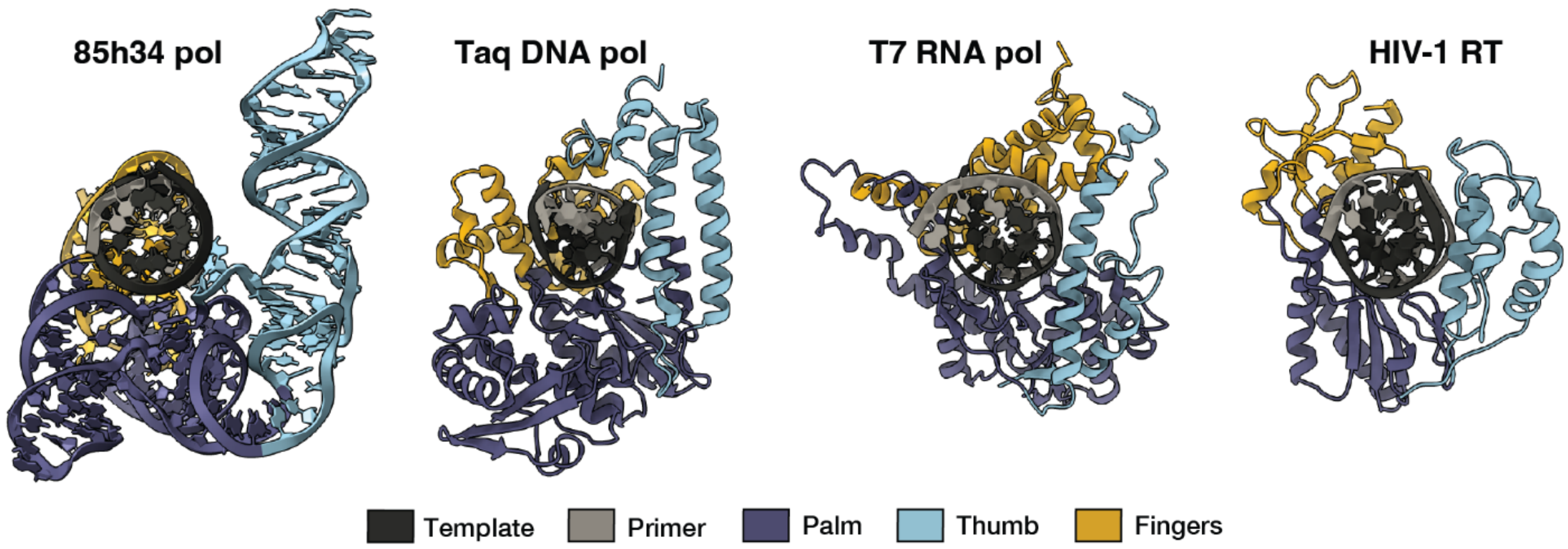

Fig. S8.

Structural convergence of the ribozyme replication complex with protein polymerases. The 85h34 replication complex structure is contrasted with protein holoenzyme complexes, including Taq DNA polymerase PDBID 1TAU (*48*), T7 RNA polymerase PDBID 1S76 (*30*), and HIV-1 reverse transcriptase PDBID 2HMI (*49*).

Data collection		*Holo*-85h34 replication complex					
Microscope		Titan Krios G3 (Thermo Fisher)					
Detector		K3 (Gatan)					
Voltage (source)		300 kEV					
Magnification		105000X					
Pixel size		0.83 Å					
Total dose		50.0 e–/Å2					
Exposure time		2.7 seconds					
Frames		50					
Dose rate		0.69 e–/pixel/frame					
Number of frames		50					
Defocus range		–0.7 to –2.5 µm					
Automation software		EPU					
Movies collected		34997					
**Data Processing**		**Consensus map**		**Subclass 2**		**Subclass 7**	
Data processing software		CryoSPARC v4.7.0 / Relion v5.0b		CryoSPARC v4.7.0 / Relion v5.0b		CryoSPARC v4.7.0 / Relion v5.0b	
Total extracted particles		4854135		1155053		1155053	
Final particle stack		1155053		98137		97830	
Gold standard FSC		2.84 Å		2.92 Å		2.94 Å	
3D FSC range		2.92–2.45 Å		3.07–2.88 Å		3.19–2.90 Å	
Sampling compensation factor		0.99		0.97		0.95	
EMDB ascension		EMD-78851		EMD-78852		EMD-78853	
**Modeling**							
Software		Coot 1.2 / Phenix 2.1		Coot 1.2 / Phenix 2.1		Coot 1.2 / Phenix 2.1	
Identifier		pdb_000038JC		pdb_000038JD		pdb_000038JE	
Map-to-model FSC (masked, cut off 0.5)		2.93 Å		3.03 Å		3.03 Å	
All-atom	Clashscore, all atoms:	2.89		1.67		3.64	
Contacts	Wrong sugar puckers:	0	0.5%	0	0.0%	3	1.8%
Nucleic acid	Bad backbone:	39	16.5%	35	16.3%	69	33.6%
Geometry	Bad bonds:	0 / 5128	0.0%	0 / 5175	0.0%	0 / 4891	0.0%
	Bad angles:	0 / 7979	0.0%	0 / 8052	0.0%	0 / 7607	0.0%
	Chiral volume outliers:	0/1064	0.0%	0/1074	0.0%	0/1014	0.0%
Composition	Nucleic acid	213		221		203	
	Waters	198		155		82	
	Magnesium	14		13		13	
	Waters with clashes	9	4.5%	12	7.7%	1	1.2%

Table S1.

Cryo-EM data collection, processing, and model building statistics.

bioRxiv preprint doi: https://doi.org/10.64898/2026.08.30.748161; this version posted August 31, 2026. The copyright holder for this preprint (which was not certified by peer review) is the author/funder, who has granted bioRxiv a license to display the preprint in perpetuity. It is made available under a CC-BY 4.0 International license.

Name	R or DNA	Source	Sequence (5´→ 3´)
85h34	R	ivt	AGAGGGAAACCUCUGCCGCCGCCGCACCAAGACAAAUCUCCCCUCAG AGCCUCAGAACAUCGAAAGAUGCAGAGCAGGGAGGCUUCGGUGGAUA AUGGUGCACCACCGUUCUGAGCACGUACCCGAACGUAAAUUGACCUG ACAGAAAGGCGUAGUUAGAAUCGCACAGGUACCAUGCCCAACACAUG GCUGAC
T1	R	ivt	GACGGCGGCAAAAAACACAAGUGAUAACAACUUUCUCGCUCC
P1	R	syn	GGAGCGAGAAAGUUGUUAUC
P1fb	R	syn	fam-biotin-GGAGCGAGAAAGUUGUUAUC
T1_tem	D	com	GGAGCGAGAAAGTTGTTATCACTTGTGTTTTTTGCCGCCGTCTATAGTG AGTCGTATTACTGG
prom	D	com	CCAGTAATACGACTCACTATAG
Fwd1	D	com	GGACTAATACGACTCACTATTAGAGGGAAACCTCTGCCGCCGCCGCAC CAAGAC
Rev1	D	com	GTCAGCCATGTGTTG
85h34 plasmid	R	syn	AGAGGGAAACCTCTGTCATTGCCGCACCAAGACAAATCTCCCCTCAGAG CCTCAGAACATCGAAAGATGCAGAGCAGGGAGGCTTCGGTGGATAATG GTGCACCACCGTTCTGAGCACGTACCCGAACGTAAATTGACCTGACAGA AAGGCGTAGTTAGAATCGCACAGGTACCATGCCCAACACATGGCTGAC

Table S2.

Sequences of RNA and DNA molecules used in this study. Oligonucleotides were either synthesized in-house (syn), purchased from IDT (com), or prepared by *in vitro* transcription (ivt). The 5′ end of P1fb was modified with both fluorescein (fam) and biotin. DNA encoding the 85h34 sequence was purchased from IDT as a “Rapid Gene” pUCIDT (Kan) plasmid. Sequences in red indicate the processivity tag within the ribozyme and RNA template. Note this sequence differs in materials used for cryo-EM analysis compared to the plasmid. Complementary regions between the primer and template are shown in blue. The T7 RNA polymerase promoter sequence within the forward primer or template is underlined.
